# Dynamics of Fiber Bragg Grating Formation with Femtosecond Laser Radiation

**DOI:** 10.3390/s25196138

**Published:** 2025-10-04

**Authors:** Oleg V. Butov, Dmitrii V. Przhiialkovskii, Eugeny D. Chubchev, Alexey B. Pnev

**Affiliations:** 1 Kotelnikov Institute of Radioengineering and Electronics of RAS, Moscow 125009, Russia; dvprz@yandex.ru; 2 Scientific Education Center Photonics and IR Techniques, Bauman Moscow State Technical University, Moscow 105005, Russia; pniov@bmstu.ru; 3 Dukhov Research Institute of Automatics, Moscow 127030, Russia; chubchev.evgeniy@physics.msu.ru

**Keywords:** Fiber Bragg Grating, femtosecond laser radiation, point-by-point inscription, multi-pass inscription, induced refractive index

## Abstract

This manuscript presents a study on the dynamics of fiber Bragg grating formation using femtosecond radiation in the point-by-point inscription regime. By employing a multi-pass inscription technique, the dynamics of the photoinduced formation of modified regions (strokes) were investigated through an analysis of the evolution of the Bragg grating’s parameters. The results demonstrate a decrease in the average effective refractive index during the grating inscription. This study highlights the complexity of the structural transformations induced in the optical fiber core material by femtosecond laser radiation.

## 1. Introduction

In recent decades, fiber optic technology has been rapidly developing and has become an increasingly integral part of the modern world. One of the key elements of fiber optics is the fiber Bragg grating (FBG), the creation of which was made feasible by the discovery of the photosensitivity phenomenon [1]. The main property of an FBG is its ability to reflect an optical signal propagating along the fiber within a narrow spectral range. This property allows FBGs to be used as narrow-band optical filters, fiber laser mirrors, dispersion elements, and sensors for physical quantities [1,2,3,4,5,6,7].

Currently, there are two fundamentally different approaches to FBG fabrication. The first group comprises methods that utilize the interference of the inscribing radiation. The interference fringes of the laser beam induce a periodic refractive index modulation in the fiber core through the material’s photosensitivity [8,9,10,11]. These are the most well-established methods used to manufacture the overwhelming majority of FBGs. Furthermore, UV inscription through a phase mask is considered the standard industrial technique. The main disadvantage of these methods is their dependence on the photosensitivity of the fiber core material. Most standard optical fibers, including those used in telecommunications, exhibit extremely low photosensitivity, which is insufficient for efficient inscription with standard UV lasers. This necessitates the use of techniques to artificially enhance photosensitivity, such as loading the fiber with molecular hydrogen [12]. However, this not only complicates the FBG fabrication process but can also adversely affect the gratings’ performance characteristics [13,14,15].

An alternative approach to FBG fabrication is direct inscription using lasers with ultrashort pulses, typically femtosecond (fs-lasers). Direct inscription methods include Point-by-Point [15,16,17,18,19,20,21], Line-by-Line [19,22,23], Plane-by-Plane [24,25], and Core Scanning [26,27] techniques. These methods are united by the principle of a step-by-step formation of the grating structure: the inscribing radiation is focused to a single point, line, or plane, and a predefined periodic structure is created by moving the focus relative to the fiber core. The use of fs-lasers overcomes the limitation of low fiber photosensitivity. Due to the high peak intensity that induces multiphoton absorption, femtosecond radiation modifies not only photosensitive impurities (as in classic UV-laser inscription) but also the regular structure of the silica glass itself.

The interaction between UV radiation and fiber materials has been studied in depth. The main mechanisms causing changes in the refractive index have been described in detail, making the process of fiber Bragg grating inscription controllable and highly predictable [2,28]. In contrast, the formation of FBGs via direct fs-inscription methods involves different underlying mechanisms. Firstly, only one local point is exposed at a time, unlike interference methods where the entire grating (or a significant part of it) is inscribed simultaneously. Secondly, the peak intensity of fs-radiation is extremely high; when focused, the power density can reach 10^13^–10^14^ W/cm^2^ [29,30]. At such high intensities, the core material is modified through multiphoton processes. Consequently, even when using visible or IR radiation with a photon energy of 1.1–3.2 eV, it is possible to affect not only impurity germanium-associated defects (with an excitation energy of 3.7–6.7 eV [31,32,33]) but also the fundamental absorption region of silica glass, which is determined by its bandgap of about 8.5–9.3 eV [34,35]. Thus, for most fs-sources, Bragg grating inscription involves 3- to 5-photon processes [36]. A significant drawback of this approach, however, is the strongly nonlinear dependence of the inscription dynamics and the final result on fluctuations in the laser’s power or pulse duration.

It is also important to consider that the quality of laser beam focusing is critical for direct inscription. In the Point-by-Point method, the characteristic size of an induced modification is significantly smaller than the diameter of the fiber core, typically about 0.3–0.5 μm in length. The most pronounced change in the refractive index occurs at the very center of the focal region, arising primarily from a density change [37,38,39]. At the same time, additional mechanical stresses may develop in the peripheral zones of the focal region and beyond. Consequently, mechanisms of different natures are responsible for the refractive index change in these respective areas.

### 1.1. Physical Mechanisms of FBG Inscription

For direct inscription methods, femtosecond lasers operating in the near-IR or visible range are commonly used as radiation sources, with typical pulse durations ranging from several tens to several hundreds of femtoseconds. As noted previously, the primary phenomenon governing the interaction between the fs-radiation and the fiber material is multiphoton absorption. The absorption of radiation by structural defects (e.g., germanium-related or silicon oxygen-deficient centers), which are responsible for “classic” photosensitivity, can induce changes similar to those observed under ultraviolet radiation [40,41]. Furthermore, unlike the single-photon absorption mode typical of UV lasers, femtosecond irradiation has a high probability of inducing structural modifications directly in the regular network of silica glass.

A second phenomenon inherent in the interaction of ultrashort laser pulses with the glass network is avalanche ionization [42,43,44,45]. After valence electrons are excited into the conduction band, they remain exposed to the intense incident pulse with its extremely high electromagnetic field. These electrons can be rapidly accelerated, gaining sufficient energy to excite secondary carriers, thereby initiating an avalanche ionization process. This leads to the formation of a solid-state plasma at the focal point [45], characterized by an extremely high electron temperature (on the order of thousands of degrees) with minimal heating of the bulk material.

The balance between multiphoton absorption and avalanche ionization depends on the radiation properties, specifically the wavelength, intensity, and pulse duration. Studies show that at relatively moderate pulse energies and short durations, the interaction results in an isotropic refractive index (RI) change at the focal point [42,43]. In contrast, at high energies and longer durations, avalanche breakdown prevails, leading to the formation of microcavities. For intermediate pulse durations and energies, a state can arise where avalanche breakdown is only partial: a solid-state plasma is effectively formed, but its energy is insufficient to cause a microexplosion. Upon cooling, this can result in the formation of microgratings and nanosized cavities (a porous glass layer) [46,47]. Consequently, the RI modulation becomes anisotropic. However, due to the small size of the modified region, this anisotropy does not significantly affect light propagating along the fiber and can be approximated as a homogeneous defect with an average refractive index.

Traditionally, changes in the refractive index have been attributed to several mechanisms: the activation of various material defects, the photorefractive effect, phase transformations (such as melting, crystallization, and re-solidification), the formation of microstructures differing from the original material, and the development of mechanical stresses [21,34,42,48].

Clearly, the described situation is further complicated by several factors: the characteristics of the inscription radiation focusing by specific optical elements, the initial photosensitivity of the fiber material, the presence of defects and inhomogeneities, and the strong dependence of multiphoton absorption processes on the duration and intensity of the radiation. These factors can significantly affect the final outcome. Consequently, the direct inscription of FBGs using ultrashort pulse lasers remains a challenging task from both technical and scientific perspectives.

### 1.2. Relevance

The primary objective of this study is to elucidate the mechanisms of fiber Bragg grating formation by analyzing the evolution of key grating parameters during the inscription process using the Point-by-Point method. The enabling tool for this investigation is the multi-pass inscription technique described in [49], which is an enhancement of the well-known Single-Shot Point-by-Point method. This approach allows the fiber core material at the focal points to be exposed to multiple laser pulses. Furthermore, it eliminates potential thermal effects associated with a high pulse repetition rate, as the system has sufficient time to relax completely between passes.

The multi-pass method effectively turns the Bragg grating itself into a sensitive probe for monitoring laser-induced changes in the material parameters. By observing the dynamics of key grating parameters, such as the reflectivity and Bragg wavelength, it is possible to deduce changes not only in the refractive index modulation but also in the average effective refractive index—something that cannot be determined using the standard single-pass method. This capability facilitates the development of hypotheses regarding the underlying processes and helps identify the key mechanisms responsible for the formation of the Bragg structure in the optical fiber.

It should be noted that several publications describe a hybrid FBG inscription approach using femtosecond lasers in an interference setup, which also enables in situ monitoring of grating parameters [50,51,52]. However, in the Point-by-Point method, the structural modifications typically occur at higher peak intensities and lower pulse repetition rates. Consequently, the underlying inscription mechanisms in these two approaches may differ significantly.

## 2. Materials and Methods

**Figure 1:**
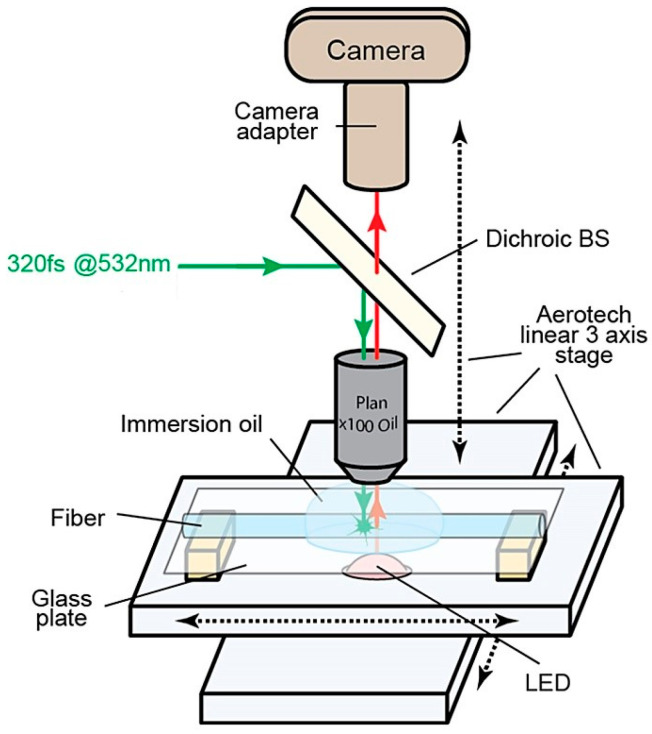
Experimental setup for fs point-by-point Bragg grating inscription.

The Bragg gratings studied in this work were inscribed using a multi-pass (multi-iteration) technique, described in detail in [49]. The experimental setup is shown in Figure 1. The inscription was performed with the second harmonic radiation (532 nm) of an ytterbium femtosecond laser (FL300, OptoSystems, Moscow, Russia) with a pulse duration of approximately 320 fs. The gratings were fabricated by translating the fiber along its axis at a constant speed relative to the laser focus. Each grating element (stroke) was formed by a single laser pulse per iteration. The fiber position and translation speed were controlled by high-precision Aerotech nanostages. For the second and subsequent iterations, the laser pulses were precisely aligned with the previously inscribed areas through synchronized operation of the laser and the nanostages.

The short wavelength of the laser radiation allowed it to be focused to a small spot (less than 0.3 μm), enabling the inscription of first-order Bragg gratings with a period of 535 nm for our experiments. The grating period was determined by the translation speed (approximately 1.07 mm/s) and the laser pulse repetition rate (2 kHz). Each grating had a length of 5 mm, resulting in an inscription time of less than 5 s per iteration.

To investigate the dynamics of FBG formation in the multi-pass regime, experiments were conducted with varying inscription laser power. Bragg gratings were inscribed in fibers with different levels of initial photosensitivity: standard telecommunication fiber Corning SMF-28 (with a low-doped germanosilicate core), a fiber with an undoped silica core (SiO_2_), and a photosensitive fiber co-doped with germanium and boron (GeB). Using fibers with different compositions allowed us to assess the impact of initial photosensitivity on the femtosecond FBG inscription process. The key parameters of these fibers are listed in Table 1.

## 3. Results and Discussion

The key parameters of a first-order fiber Bragg grating, such as the Bragg wavelength and the reflection coefficient (*R*), are determined by Equations (1)–(3) [2]:

(1) $$\lambda_{B}=2n_{eff}\Lambda$$

where *n_eff_* is the effective refractive index of the structure, Λ is the structure’s period.

The grating’s reflection will be determined by the Formula (2):

(2) $$R={tanh}^{2}\left(\kappa_{B}L\right),$$

where *L* is the length of the Bragg structure, *κ_B_*—coupling coefficient, also known as the grating strength, determined by Formula (3) [4]:

(3) $$\kappa_{B}=\frac{\pi \Delta n_{mod}\eta }{\lambda_{B}}$$

where Δ*n_mod_* is the averaged value of the refractive index modulation along the fiber induced by the laser radiation and *η* is the overlap integral, which determines the fraction of the energy of the propagating mode overlapping with the cross section of the photoinduced defect.

**Figure 2:**
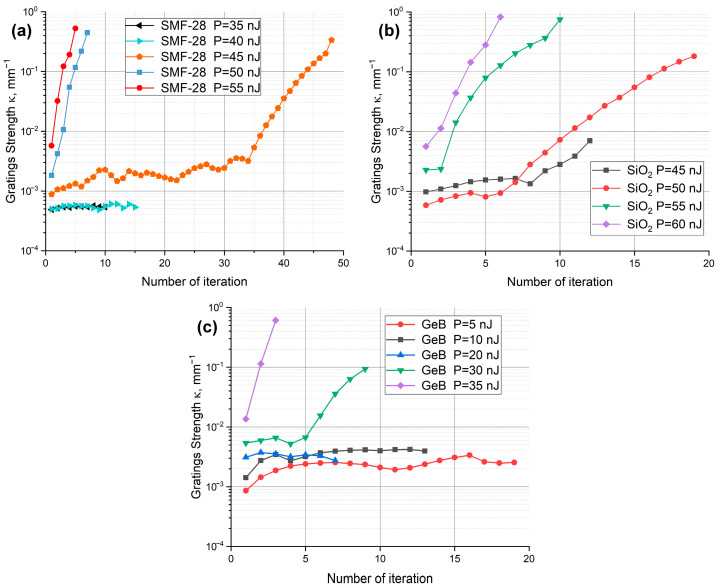
Dynamics of changes in the strength of Bragg gratings (3) during inscription depending on the laser pulse energy: in Corning SMF-28 fiber (**a**), pure-silica-core fiber SiO_2_ (**b**), photosensitive fiber GeB (**c**).

As noted previously, the peak power level of the laser radiation is a critical parameter in FBG inscription. At low power levels, effects associated with photosensitive centers that have a lower activation energy than the bandgap of silica glass become more pronounced. The inscription effect at low energies is weak, and the Bragg wavelength shift is negligible. The reflectivity was measured using a Micron Optics SM125-200 Bragg interrogator. The corresponding experimental results are presented in Figure 2.

Figure 2 shows the dynamics of grating inscription in three different fibers: Corning SMF-28 with a relatively low level of germanium core doping (Figure 2a), a pure-silica-core fiber (SiO_2_, Figure 2b), and a photosensitive fiber with a high concentration of germanium and boron in the core (GeB, Figure 2c). As seen in the graphs, the inscription dynamics in the first two fibers are similar in both characteristics and required pulse energy. Generally, the dynamics can be divided into three stages: initial rapid formation of index modulation leading to a weak Bragg grating, followed by several irradiation cycles with no measurable grating growth, and a potential subsequent increase in grating strength at higher pulse energies (above 45–50 nJ).

The similarity of the first two graphs suggests similar structure formation mechanisms. However, the threshold pulse energy for observable grating formation is lower in SMF-28 due to the presence of photosensitive centers, albeit in low concentration. At low, near-threshold energies, multiphoton absorption affects individual bonds of these centers. At higher energies, it affects bonds in the regular glass network, forming silicon and germanium E′-centers. These centers introduce new absorption bands, which, according to the Kramers–Kronig relations, lead to an increase in the refractive index. This results in a weak grating with a fixed saturation level, determined not only by the dopant concentration but also by the ratio of the size of the induced modifications to the mode field diameter, which is somewhat larger in the SMF-28 sample. Consequently, the observed saturation level for this sample is proportionally lower than in the pure-silica-core fiber, which has a mode field diameter of approximately 5.9 μm compared to 10.4 μm for SMF-28.

Multiple exposures of the irradiated areas increase the probability of exciting multiple bonds associated with the same silicon atoms and impurity defects. Further irradiation can lead to the formation of nanogratings (density-modulated structures) and nanocavities [47,53].

The energy and duration of a single laser pulse are insufficient to trigger avalanche ionization and form a microcavity throughout the entire focal volume. However, it is quite probable that local nanopores form due to energy transfer to already excited bonds, resulting in a porous glass structure. This hypothesis is supported by the gradual increase in grating strength with each subsequent iteration. In contrast, if avalanche ionization occurs and a microcavity is formed, subsequent laser exposure—while capable of further altering it—would exhibit sharply nonlinear behavior.

The final outcome of these transformations is a highly reflective Bragg grating. It is noteworthy that the second stage of grating formation is observed in both fiber types within the same pulse energy range, further confirming the dominant role of processes occurring within the regular silica glass network. A similar effect is observed at higher intensities, with the key difference that the probability of exciting multiple atomic bonds increases significantly from the first pulse. Additionally, the transfer of extra energy to excited electrons at the focal point leads to the formation of a solid-state plasma, facilitating the rapid formation of nanogratings, nanocavities, and a highly reflective Bragg grating within a small number of iterations.

A different behavior is observed in the third sample, which has a high concentration of germanium and boron in the core. In this case, the required inscription energy is significantly lower. For the first two fibers, the threshold energy for grating inscription was no less than 25–45 nJ per pulse, whereas for the third sample, inscription is observed at energies as low as 5 nJ. At 35 nJ, a highly reflective grating is rapidly formed. This indicates that the formation of the Bragg structure in this fiber is dominated by numerous photosensitive impurity centers with low excitation energy (relative to the bandgap of silica glass) [33]. Consequently, these centers can be excited with a lower order of multiphoton processes, which explains the low activation energy threshold.

A further increase in pulse energy raises the probability of exciting multiple bonds within photosensitive centers and the regular glass network, as well as the likelihood of plasma formation. This triggers processes similar to those observed in the first two samples, with the key difference that the required activation energy is also lower.

**Figure 3:**
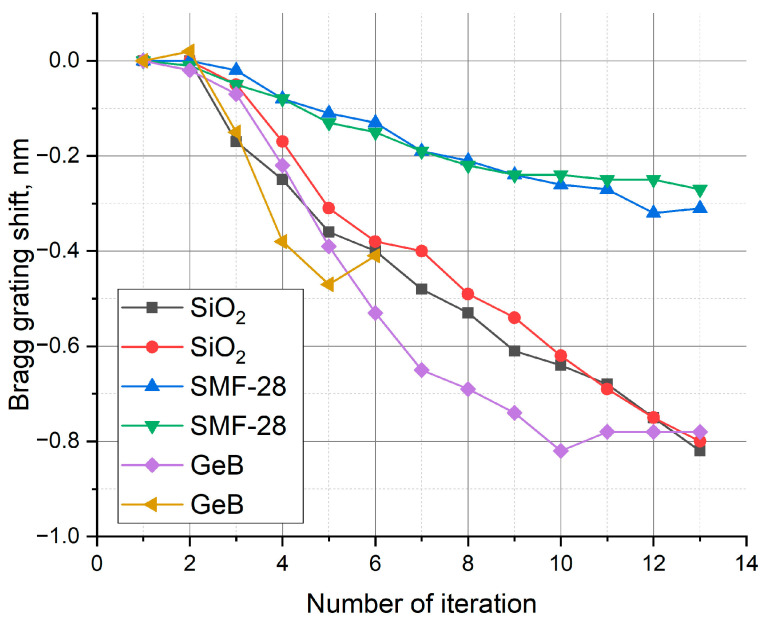
Bragg wavelength shift during the FBG inscription process in different fiber samples with the same (70 nJ) energy of the inscribing laser radiation pulses. Each type of fiber was tested twice.

The Bragg wavelength is the most important parameter characterizing the grating and the processes occurring during its inscription. While the reflectivity (or grating strength) is proportional to the amplitude of the effective refractive index modulation along the fiber, the shift in the Bragg wavelength reflects changes in the average effective refractive index. Previous work [49], which proposed the multi-pass FBG inscription method, reported a small blue shift in the Bragg wavelength that correlated with an increasing number of inscription iterations. This reproducible effect was observed in all samples studied in the present work (Figure 3).

Since the grating period remains constant during inscription, a change in the Bragg wavelength, according to Equation (1), indicates a change in the average effective refractive index. The inscriptions were performed at a pulse energy of 70 nJ, resulting in a Bragg wavelength shift ranging from −0.3 nm to −0.8 nm across different samples, corresponding to a decrease in the refractive index. This result is fully consistent with the hypothesis of a porous structure forming in the laser-exposed region, where the creation of microvoids would lead to a reduction in this parameter.

However, the formation of nanopores should lead to a redistribution of the glass density, causing compaction of the surrounding material. As shown in [37,38,39], the relationship between the refractive index and density is approximately linear over a wide density range (at least up to 20% [38]), making a linear approximation sufficient for estimating the refractive index change. Within this linear model, density redistribution at a constant material volume should not alter the average (integrated) refractive index and thus cannot account for the observed blue shift in the Bragg wavelength during FBG inscription.

It is important to note that the power distribution of radiation propagating along the fiber core is non-uniform and is described by Bessel functions [54]. Consequently, the power density is highest at the core center and decreases with distance from the fiber axis. Accordingly, changes in the refractive index at the core center have a stronger impact on the propagating mode than changes at the periphery. Therefore, even with no net change in the integrated refractive index over the entire glass volume, the effective refractive index experienced by the optical mode can still change.

**Figure 4:**
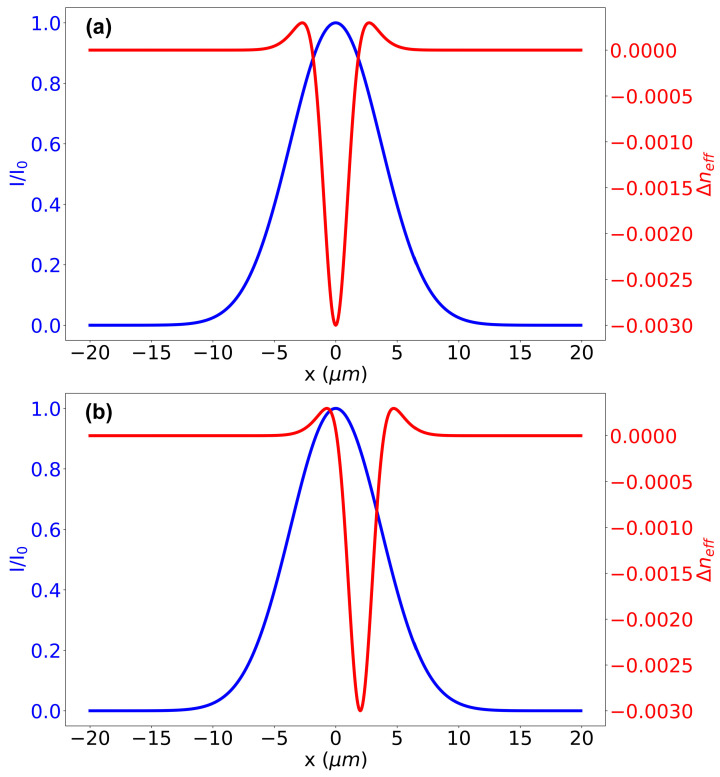
Radial redistribution of the silica glass density (expressed as the refractive index, Δ*n_eff_*) around the laser radiation focal point (red curve) and the corresponding fiber core mode field distribution (blue curve). (**a**) Focal point located at the fiber center. (**b**) Focal point shifted 2 μm from the center.

This effect is illustrated in Figure 4a, where the mode field distribution acts as a weighting function for the radial changes in the refractive index. The most significant effective changes occur where the mode field intensity is highest. In this case, the dominant effect is a negative change in the refractive index.

It is also evident that a smaller mode field diameter, for the same size of photoinduced modification, will amplify this effect. This can explain the more substantial blue shift in the Bragg wavelength observed in fibers with smaller cores and, consequently, smaller mode field diameters. This conclusion is supported by an analysis of the data presented in Figure 3. At the same inscription energy, the blue shift differs among the samples: for Corning SMF-28 fiber, it is only 0.3 nm, whereas in the undoped silica core fiber and the photosensitive fiber, it exceeds 0.8 nm. Notably, the mode field diameter in these latter samples is approximately 5.3–5.9 μm, compared to 10.4 μm for SMF-28.

Furthermore, the change in the effective refractive index experienced by the propagating mode depends on the position of the laser focal point relative to the core center, all other factors being equal. As shown in Figure 4b, the impact of the negative refractive index change diminishes as the focal point moves away from the fiber axis, while the contribution from the positive index change in the peripheral region of the modified zone becomes more pronounced.

**Figure 5:**
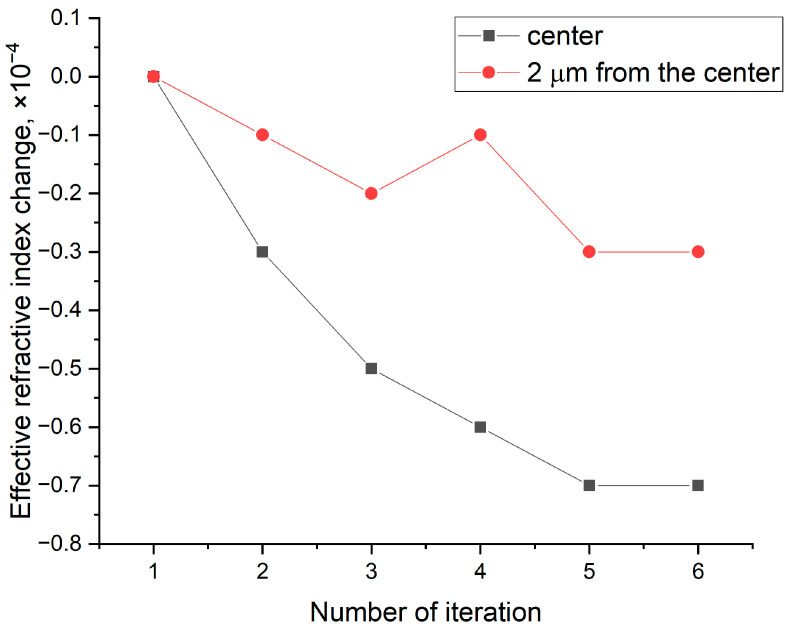
Effective refractive index change during axial grating inscription (black curve) and for a 2 μm displacement from the center (red curve).

This hypothesis is experimentally verified in Figure 5, which shows the evolution of the effective refractive index for gratings inscribed under identical conditions but with different focal point positions. In the first sample, the grating was inscribed with the focal point at the fiber center, while in the second, it was displaced by 2 μm. The effective refractive index change in the second case is approximately half that of the first, a result supported by numerical modeling.

For instance, assuming a characteristic refractive index change of −3 × 10^−3^ at the exposure center, an effective modified region radius of 1 μm, and a mode field diameter of 10.4 μm for SMF-28, the calculated change in the effective refractive index is −0.7 × 10^−4^ for axial inscription and −0.45 × 10^−4^ for a 2 μm displacement. These values show good agreement with the experimental results, within the measurement error.

## 4. Conclusions

This study investigates the dynamics of fiber Bragg grating inscription via a multipass point-by-point method using femtosecond laser radiation at varying power levels in optical fibers with different compositions and initial photosensitivity. A model is proposed for the complex structure of the induced grating element (stroke), which postulates a negative refractive index change at the center of the focal region. This change is attributed to the formation of a porous glass structure that reduces material density. In contrast, a zone of material compaction, which contributes positively to the refractive index, is observed at the periphery of the focal region. The overall negative change in the effective refractive index is explained by the energy distribution profile of the fiber’s core mode. The most significant contribution to the effective refractive index originates from the central part of the fiber, which coincides with the region of maximum energy density in the core mode.

## Figures and Tables

**Table 1:** The key parameters of the fibers used in the experiments.

Fiber Type	Co-Doping	Core/Cladding RI Difference, 10^−3^	Core Diameter, μm	Mode Field Diameter, μm
SMF-28	Ge	5	8.5	10.4
SiO_2_	-	16	5.0	5.9
GeB	Ge, B	17	4.0	5.3

## Data Availability

Data are contained within the article.
